# A Supervised Approach to Predict the Hierarchical Structure of Conversation Threads for Comments

**DOI:** 10.1155/2014/479746

**Published:** 2014-02-11

**Authors:** A. Balali, H. Faili, M. Asadpour

**Affiliations:** School of ECE, College of Engineering, University of Tehran, P.O. Box 515-14395, Tehran, Iran

## Abstract

User-generated texts such as comments in social media are rich sources of information. In general, the reply structure of comments is not publicly accessible on the web. Websites present comments as a list in chronological order. This way, some information is lost. A solution for this problem is to reconstruct the thread structure (RTS) automatically. RTS predicts a semantic tree for the reply structure, useful for understanding users' behaviours and facilitating follow of the actual conversation streams. This paper works on RTS task in blogs, online news agencies, and news websites. These types of websites cover various types of articles reflecting the real-world events. People with different views participate in arguments by writing comments. Comments express opinions, sentiments, or ideas about articles. The reply structure of threads in these types of websites is basically different from threads in the forums, chats, and emails. To perform RTS, we define a set of textual and nontextual features. Then, we use supervised learning to combine these features. The proposed method is evaluated on five different datasets. The accuracy of the proposed method is compared with baselines. The results reveal higher accuracy for our method in comparison with baselines in all datasets.

## 1. Introduction

In recent years, interactive online websites such as weblogs, discussion boards, and news websites have grown in popularity, so these online threads have become valuable source of information. This information is obtained by interaction among users who create, share, and exchange information and ideas on various topics such as politics, economy, society, and environment. People tend to express their ideas and opinions in public and online [1]. Millions of web-users spend hours a day on these online threads in order to read news and articles, write their opinion and discuss with each other.

Nowadays, most of the websites use content management systems that allow them to receive feedback from visitors and collect their comments. These comments are publicly showed to the visitors, usually after confirmation by a moderator. These comments are showed mainly in chronological order (sometimes in reverse order), that is, when a user posts a comment, it is appended to the end of the list.

Although content management systems nowadays allow nested comments (in hierarchical order), due to space problem showing the complete hierarchy might not be possible. That is why in many websites, either the nested hierarchy is not shown or the depth of the hierarchy is limited to something like 3 or 4 levels. This problem makes following the discussions very difficult and time consuming.

Here, we try to automatically reconstruct the thread structure (RTS). We try to build a semantic tree where nodes of the tree (except the root node) are comments and edges that specify which comment is in reply to which comment. The root of the tree is the main article.

Blogs and online news agencies are very important for the opinions they receive from visitors. Also, since they cover various events of our social life and many people with different views participate in the arguments, they are valuable source of information for researchers as well.

We divide the discussion threads into interrogative and declarative threads. Discussion threads in which users write a question and the other users try to answer it like forums, chats, and emails [2–7] are interrogative threads. Threads like blog comments and comments that discuss news articles do not fit to a question-answer format. We call them declarative.

Valuable studies have been done on RTS in interrogative threads [2–7]. However, due to the difference between these threads, they are not effective enough on declarative threads. In this paper we focus on declarative threads and try to devise an effective RTS method for them. In summary, the RTS task that we are doing here is suitable for the following websites:

- websites that show comments in a list based structure in chronological order. This way of showing comments is prevalent [2–4], for example, Reuters (http://www.reuters.com/) and ABCNEWS (http://www.abcnews.com/) which are listed, respectively, as the 301th and 462th most popular websites in global based on the Alexa's traffic rank (Alexa—The web information company, http://www.alexa.com/);
- blog service providers who do not support hierarchical structure for their comments such as Blogfa (http://www.blogfa.com/) which is listed at the 137th most popular website globally and 3th popular website in Iran after Google and Yahoo;
- websites that cannot support a tree-like reply structure with more than some levels, due to the limitation of space on pages such as Facebook, Fin24 (http://www.fin24.com/), and Skysport (http://www.skysports.com/);
- websites that have been created from content management systems that do not support nested comments, for example, those who have designed the template of their sites based on an old version of Wordpress (before 2009) and have not changed it.

RTS can be beneficial in many applications. To name a few, it is useful for facilitating search and finding of the user's favorite content in a large volume of comments and for improving retrieval accuracy [2, 8, 9], identifying users who have the ability to answer the questions [10], isolating discussions related to specific subtopics [11], understanding the online user's behavior [12], facilitating the follow of the actual conversation stream in threads [4], conversation summarization which include both the initiation and response as a coherent unit [13], automatic question, and answer detection [13, 14], and finding the reply relations among comments to be used in other tasks like topic detection.

Another benefit for RTS is in topic detection on comments. Usually, comments are short in length and this makes the topic detection task quite difficult. Usually topic of comments is similar or related to topic of its parent and its children. So, knowing the hierarchy can help us to provide extra information in order to enhance topic detection. This can be considered as a sort of word expansion.

In this work, we propose a method to automatically reconstruct thread structure and organize comments into a tree-like structure by considering information about authors, content, date, and time of the post. A set of relevant textual and nontextual features are defined. Then, a learning algorithm based on ranking SVM model is used to learn a proper model that is exploited to identify the reply relation between a root and a set of comments. In other words, a set of comments is fed into the trained model to determine if there is any relation between them or not. The proposed RTS method is called SLARTS (a Supervised Learning Approach to Reconstruct Thread Structure). We combine our knowledge and technics from Information Retrieval, Natural Language Processing, Machine Learning, and Social Network disciplines.

The main contributions of this work are as follows.

- The focus of this paper is RTS task on declarative threads in blogs, online news agencies, and news websites on which few studies have been carried out.
- We describe and show the differences between the declarative and interrogative threads. To better illustrate the differences, we used Apple discussion forum and compared it with declarative datasets.
- We propose a supervised approach to RTS task, namely, SLARTS, based on ranking SVM model using novel textual and nontextual features which are related to declarative threads.
- The proposed method is tested on 5 datasets in different languages: one in Persian, one in Russian and three in English. In three of these websites the comments are confirmed by moderators. Some of these websites present comments as user posts. All datasets consist of lots of reply structures among comments. The reply structures of comments in ground truth come from real structures that are created by users and they are addressed by reply tags in our datasets.
- In order to better evaluate our method, some evaluation metrics that are proposed in the previous works have been modified and an evaluation metric is proposed to RTS task which is appropriate for declarative threads.
- The evaluation results reveal higher accuracy in comparison with the baselines methods in all datasets.

This paper is an extension of the work we published in our conference paper [15]. We have defined new textual and nontextual features to improve the accuracy of the proposed method. We also evaluate our method on more datasets with new measures for accuracy.

The rest of this paper is organized as follows. In Section 2, we describe related work. It is followed by problem definition in Section 3. We explain the proposed method and features used for RTS task in Section 4. In Section 5, the experiments, datasets, evaluation metrics, and experimental results are shown and described. Finally, we discuss the results and conclude with the valuable information which can be extracted by visualizing the reply relations structure.

## 2. Related Work

In this section, we cover previous works on RTS task and then we will investigate various issues around threads in online platforms.

### 2.1. Thread Detection

Thread detection task, which sometimes is called topic detection, should be accomplished as preprocessing for the RTS task. In this task, all comments are split into a number of threads. After that RTS could be exploited to discover the tree structure of the threads [5, 10, 16].

The thread detection task is not necessary for declarative threads, since usually all comments are related to the root. Based on this, all comments can be considered in just one thread. Consequently, we focus only on RTS task in this study.

### 2.2. RTS Task

There are very few studies in the literature that directly address the problem of RTS task for comments in declarative threads. In general, RTS task can be done either in supervised or unsupervised manner. In unsupervised methods, the relation between comments is weighted by using text similarity measures. Then, they are adjusted using other metrics such as time distance and position in chronological order. Finally, the relations whose weights are higher than a predefined threshold are selected as the parent-child relations [11, 17]. Lin et al. [18] proposed a method which computes the similarity between each post and previous posts to find candidate parents. Then the post with the highest score is chosen as the parent candidate. If the parent candidate is not similar enough to the child comment, the candidate parent is assumed to be a new discussion branch of the thread.

In supervised methods, the existence of relation between two messages is determined using a supervised learning algorithm [2, 4, 19]. In these methods, a set of features is defined and weighted using a training set. Then, the trained model is used to discover the comment relations in the test data, using extracted features.

The methods proposed in [4, 19] are supervised, in which a set of simple features and a classifier are used. The features are divided into two groups. The first group is called structural or nontextual features such as time information, reply distance, and author's name. The next group of features is called semantic or textual features such as sentences type and similarity among comments.

Seo et al. [2] proposed a learning technique that exploits the hierarchical structure of replies in forums or emails. They introduced a structure discovery technique that uses a variety of features to model the relations among posts of the same thread. In fact their method is the most similar to ours. However, we focus on blogs and news agencies while they have worked on forums and emails. The existence of quoted texts in forums and emails makes RTS task easier than our case.

Wang et al. [3] proposed a probabilistic model as a supervised structure learning problem to predict the dependency among the posts within one thread on forums based on the general conditional random fields. Their method is based on various kinds of features. The features described the interactions in both posts and authors. The weights for the designed features are estimated in a supervised manner. Similar to previous work, Wang et al. [20] proposed a discriminative probabilistic model which can handle both local features and knowledge propagation rules.

The only existing work on RTS task in declarative threads has been proposed by Schuth et al. [19]. This work focused on RTS task in online news agencies threads. They used several features to detect authors' name in the comments' text. Then, the features are combined by tree-learner algorithm and eventually a classifier detects relations among comments and the root. Since, there are many comments that do not refer to any author's name in our case, this method does not have a good accuracy.

There are also some related works on other types of data such as email data [6, 7, 21]. However, some specific features exist in those environments that are not applicable here. For instance, some extra information about message's recipients like “To/CC” tag in email data or any information about the affiliation of message's author like signature is exploited to improve reconstructing conversation threads in email data.

### 2.3. Thread Structure Analysis

Some works have been done on analysis of the social media emerging from the user comment activity [22, 23]. The messages boards such as Slahsdot and Reddit publish frequently short news posts and allow their readers to comment on them. The works proposed in [24, 25] focused on these threads. They explored the structure and topical hierarchies of comment threads to gain a deeper understanding of the users' behaviour that allow these types of user-powered websites to better operate. Laniado et al. [26] analyzed the structural properties of the threads on Wikipedia pages to extract and study different kinds of interactions. Wu et al. [1] introduced a model to explain the human view and reply behaviors in the forum which are helpful for discovering collective patterns of human behaviors. They found that view and the reply behaviours have a form of a power-law distribution [1, 27].

### 2.4. Detection of Initiation-Response Pairs

The works proposed in [13, 14] focused on detection of initiation-response pairs such as the question-answer and assessment-agreement relationships. Initiation-response pairs are pairs of utterances that the first pair part sets up an expectation for the second pair part [13]. Wang et al. [14] introduced a list of the dialogue act labels for edges. A dialogue act label such as answer-answer, answer-question is assigned to each relation between two messages. Kim et al. [28] proposed a dialogue act tag set and method for annotating post-to-post discourse structure on forums. They used three feature sets, structural features, post context features, and semantic features, and they experimented with three discriminative learners, SVM, HMM, and maximum entropy. Andreas et al. [29] introduced a new corpus of sentence-level agreement and disagreement annotations. Two sentences are in agreement if they show the same fact or opinion.

A list of dialog act labels and an approach for modeling dialogue acts have been proposed in conversational speech [30–32]. Detection of dialogue act labels for each post is suitable for thread detection [5, 16] and finding relevant answers in forums [8].

### 2.5. Automatic Meta-Information Extraction from HTML Pages

Information of threads such as authors' name and content is usually extracted by human. This is time consuming. Some methods have been proposed to extract the main content and remove noisy information from a web page automatically [33]. Hu et al. [34] proposed an algorithm to extract all meta-information of threads from different kinds of forums without human interaction. The algorithm consists of two steps: thread extraction and detailed information extraction from thread pages.

## 3. Problem Definition

**Figure 1:**
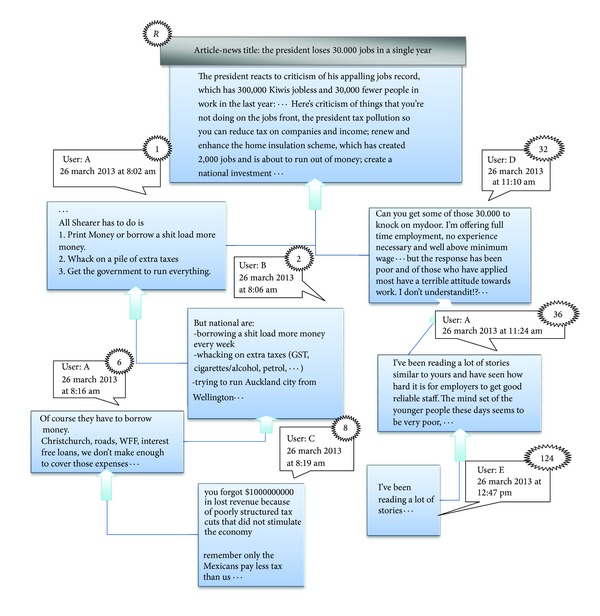
A part of a real thread from Thestandard (http://www.thestandard.org.nz) online news website.

In this section, some concepts are defined about RTS task. A *comment* is an utterance written by a user, comprising one or several sentences. A *commenter* is a person who writes comments and replies to *the root* article or the other comments. In this paper, the root is defined as the starter of a conversation which can be a news article or any other content which has been written by an author or a journalist.  Figure 1 shows a part of a real thread with 145 comments. In Figure 1, the root post is shown with label *R* and edges denote the reply relations. In a thread, a *reply comment* (which is illustrated in Figure 1 by numbered labels) responses to the previous comments or to the root item. For example, the nodes with label 1 and 32 are reply comments to the root item, which is considered as their parent. The sequences of labels in Figure 1 are in chronological order. A *thread* is a sequence of comments that starts with the root item and contains a series of reply comments which are usually related to the same topic as the root item. Each comment has a single parent and all comments are descended from the root post in a thread [24, 29]. In other words, threads are considered as a special case of human conversations [35] that consist of a set of comments contributed by two or more participants by the reply operation [36]. *The candidate set of ith comment* is a set of comments that could be considered as the parent of *i*th comment and includes comments which appear before the *i*th comment in chronological order. *The starter discussion comment* is a comment repling to the root item and it has at least one child, like comments with labels 1 and 32 in Figure 1.

*Thread detection task* means finding the cluster of comments that belong to the same topic in a given text stream without any previous knowledge about the number of threads. *Reconstructing thread structure (RTS) task* means reconstructing the reply structure on comments in a thread. This leads to construction of a tree-like reply structure [2–5] or directed acyclic graph (DAG) [11, 17, 19]. Since the threads extracted from websites have the tree-like reply structure, they are usually modeled as a tree in most papers. Also, the structure of threads can be modeled as a DAG. The information about DAG-like reply structure is mostly prepared by manually annotating the data which is a time consuming and difficult task. In addition, since the manually annotated dataset contains a small number of threads [11, 19], it cannot properly evaluate the RTS algorithms. In this paper, we assume a tree-like reply structure.

Declarative and interrogative threads are different in essence.

- Users in declarative threads mostly express their opinions or sentiments about the root post informally, while in interrogative threads, users mostly express their questions and answers in a more formal way.
- In declarative threads, the topic of the root is most likely a news article or a content reflecting the real-world events, while in interrogative threads, it is most likely a user's question.
- There is meta-information such as quotation in interrogative threads that has a great improvement on the accuracy of RTS task.
- Comments of declarative threads usually wait for moderation to be published and it usually takes some time. Moderators are not always online, they log in a few times per day, accept the sent comments, and log out. Therefore, multiple comments appear nearly at the same time. So, some features that are based on time distance [11] and position in chronological order have good performance in interrogative threads, but they are not good in declarative threads.

Declarative and interrogative threads are technically different as well.

**Figure 2:**
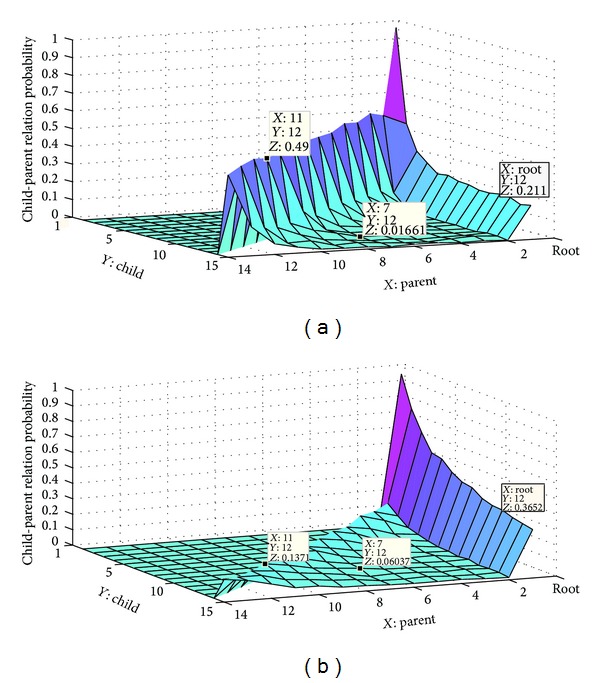
The probability of child-parent reply relations: (a) Apple discussion forum and (b) Thestandard online news agencies.

**Figure 3:**
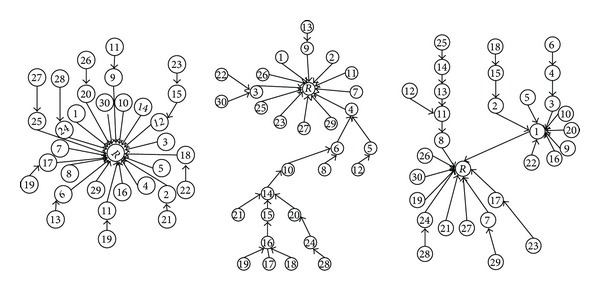
Three real threads which the nodes' label are in chronology order.

- Each comment can most likely be connected to its previous comment (1-Distance) in the interrogative threads [4]; this simple heuristic leads to great improvement in the accuracy of RTS task [3, 4], since many of posts are most likely written to answer the last question. However this heuristic cannot have a good performance in declarative threads. According to Figure 2(a), the users most likely reply to the first previous comment in Apple discussion forum. Although, Figure 2(b) shows the users most likely reply to the root in Thestandard online news agencies. The 11th comment in Apple is the parent of 12th comment with probability 0.49; however, in Thestandard, the probability is equal to 0.137.
- Figure 3 includes three real threads in Thestandard dataset which includes 30 comments and each comment is numbered and sorted in chronological order. Since, users usually express their opinions or sentiments and reply to each comment regardless of submission time and position of comments, so the structure of the replies is not predictable.
- The length of the roots text in declarative threads is usually larger than comments; thus, the length of the root or comments should be normalized in similarity measurements.

There are websites which are known as message boards such as Digg, Reddit, and Slashdot. The message boards are valuable threads to analyze users' behavior [24, 25]. However, they are not suitable to be used as training data, since they have different designs for showing the comments. These differences change the behavior of user's replies; for example, Slashdot (http://slashdot.org/) shows comments based on their scores. This causes the comments which have higher scores, to get more replies. So, we cannot create a general model appropriate for all message boards.

## 4. Method

In this section we describe our supervised approach to RTS task. First, we define some textual and nontextual features and learn a proper model to combine the features using a ranking SVM (Support Vector Machines). Then the model is employed in reconstruction of the reply structure for threads in the test data.

### 4.1. Ranking SVM

**Figure 4:**
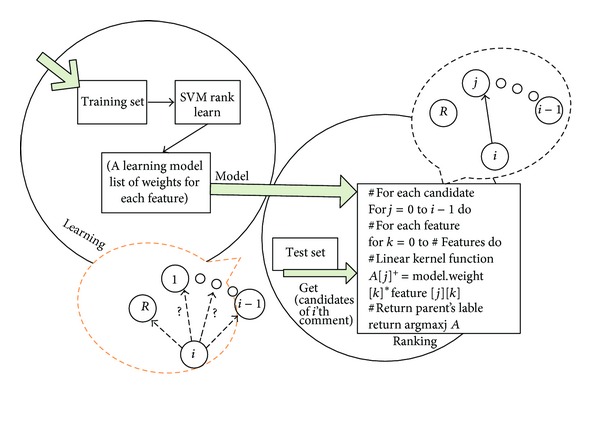
RTS algorithm.

SVM is a supervised learning method that is used for classification, regression, and ranking. The implementation we use is the ranking SVM classifier (http://www.cs.cornell.edu/people/tj/svm_light/svm_rank.html) [2, 37]. The ranking SVM classifier learns to assign a weight to the pairs of comments. The whole procedure for choosing the parent of the *i*th comment in a thread is described in Figure 4.

Since the purpose of RTS task is to predict a tree-like reply structure in a thread, RTS algorithm needs to find *N* reply relations among comments and the root, where *N* is the number of comments in a thread. Thus, RTS algorithm needs *O*(*N* ^2^) pairwise comparisons.

### 4.2. Features

The ranking SVM needs some features to be defined. In this section, we introduce eleven textual and nontextual features. Their definition is one of the main contributions of our work. It should be mentioned that about 20 percent of replies in ground truth do not have any common word with their parent. The textual features therefore are not enough and we need to define some nontextual features.

#### 4.2.1. Similarity Measurement

In order to measure the similarity of sentences, we utilize a vector space model to represent the text body of comments. A comment's text can be considered as a vector of terms, weighed by TF-IDF. TF (Term Frequency) is the number of times a word appears in a comment and IDF (Inverse Document Frequency) is computed according to the following formula:

(1) $$\begin{array}{c} \text{IDF}\;\left(w\right)=\text{Log}\left(\frac{N}{n_{w}}\right), \end{array}$$

where *N* is the total number of comments in a news item and *n*_*w*_ is the number of comments that contain the word *w*. IDF is usually extracted from the whole training data, but we have limited it to the set of comments in a thread. We believe this makes it more accurate, for example, when a word has a low frequency in the whole training data but has a high frequency in a thread.

In order to measure similarity between two comments, the stop-words are deleted first and then words of the comments are stemmed by the Porter algorithm (for English datasets). This step is not performed for Russian and Persian datasets. The common words of the two comments are extracted and their weights are calculated based on TF-IDF. Then, the final score is obtained by aggregating the weights of common words according to (2). Since the root text is usually longer than the comments, log of the product of their lengths is used in denominator:

(2) Score (C1,C2)=∑w∈C-words(1+log⁡⁡(TF(w,C1)×TF(w,C2)))×IDF(w)×(log⁡⁡(|C1|×|C2|))−1,

where *C*-words are the number of the words held in common between comments *C*1 and *C*2 and |*C*| is the length of the comment's text.

Comments are usually informal and have typo errors. Some words in two comments might be the same, but due to spelling errors, it is difficult to find this out. In order to solve this issue, we use the minimum edit distance (MED) algorithm. The minimum edit distance of two words is the minimum number of edit operations (insertion, deletion, substitution, and transposition) needed to transform one word into another [14]. The costs of insertion, deletion, substitution, and transposition are 1, 1, 2, and 1, respectively.

Two words in different comments are considered as common words if either they are exactly the same or they seem to be the same but they contain some typo errors. In the latter case, if the length of the words is bigger than five and their first two letters are the same and their edit distance is lower than 4, the two words are considered as common word. For example, two words “Beautiful" and “Beuatiful” are considered as common words.

#### 4.2.2. Authors' Language Model

**Figure 5:**
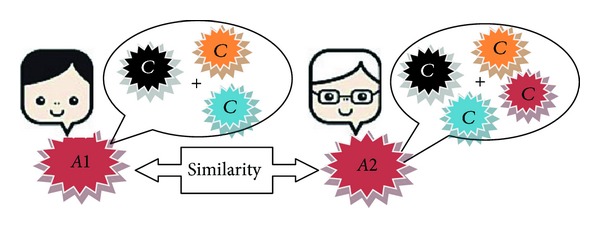
The authors' language features based on author “*A*1” and author “*A*2”.

The idea is that the commenters who talk to each other are more likely to use similar words in their comments. In order to take advantage of this feature, all comments that are related to a commenter are appended. This makes a collection of words for each commenter. If the collections of words of two commenters are very similar, they can be related to each other. In Figure 5, commenter “*A*1” wrote three comments. These three comments are appended and make a collection of words for commenter “*A*1” and then like the first feature the similarity is calculated between collection of words of commenters “*A*1” and “*A*2”. The similarity scores obtained between two commenters “*A*1” and “*A*2” are considered as this feature's score for relations between their comments.

#### 4.2.3. Prior Location

Position of comments can reveal some information about their hierarchy, for example, first comments usually have more children than the others, or comments which are located just before the *i*th comment are more likely to be its parent. In general, we would like to estimate *p*(*i* | *j*), that is, knowing that a comment is in position *j* which is the likelihood that the comment in position *i* is its parent [2]. So we calculate prior probabilities for being the parent of different positions.

**Figure 6:**
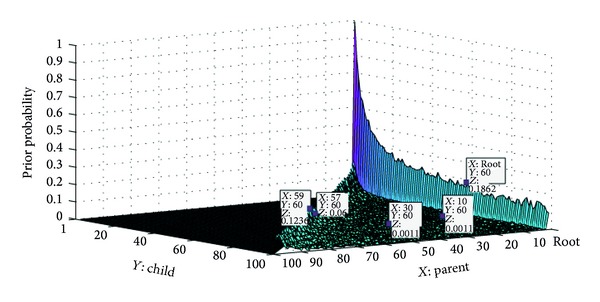
The prior probability of child-parent relation from comments 1 to 100 in Thestandard.

**Figure 7:**
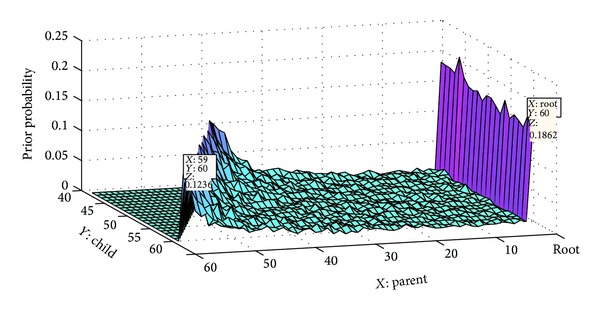
The prior probability of child-parent relation from comments 40 to 60.

To calculate prior probability for *j*, we count the number of times a comment in position *i* is the parent of *j* in the training set.  Figure 6 shows the prior probability for comments in positions 1 to 100 in Thestandard dataset. The highest prior probability belongs to the root and then to the comments which are located just before the comment. The sample points in Figure 6 show five comment's positions such as the roots 10, 30, 57, and 59 and how it is probable for 60th comment to be a child of them that the root has the most prior probability which is equal to 0.1852 and then the 59th comment which has the probability of 0.1236. Also Figure 7 shows the prior probability of child-parent relation from comment 40 to 60.

#### 4.2.4. Reference to Authors' Name

If the name of an author appears in a comment, then all his/her comments are considered as a potential candidate for being parent of that comment [19]. Sometimes a part of the complete name is referenced. Sometimes the author's name is made up of two parts and both of these parts could be used by other authors for reference. We also consider these types of references. We hold each part of the author's name and then parts which are stop-words are removed.

#### 4.2.5. Global Similarity

**Figure 8:**
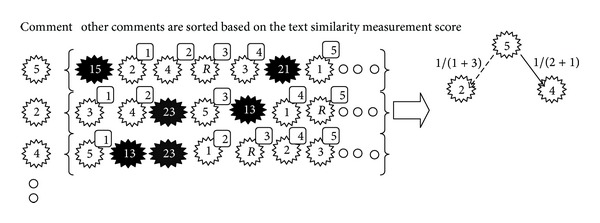
An example for global similarity features.

This feature is based on ranking the similarity of comments' text and the root's text which has a global view on the text similarity. According to this feature, if comment “*A*” has the most similarity with comment “*B*” and inversely comment “*B*” has the most similarity with comment “*A*” among other candidates, it is more likely that there is a relation between comment “*A*” and comment “*B*”. To relax this feature, the similarity measurement is calculated for each comment corresponding with all comments; then the comments are sorted based on their similarity score. For example, in Figure 8, we are looking for parent of the fifth comment. In the first step, according to Formula (2) comments are sorted based on score of text similarity measurement per the fifth comment. Comments that do not belong to candidate of the fifth comment are removed. The removed comments have been shown with black color in Figure 8. In the second step, the same as the first step, comments are sorted per each candidate of, fifth comment and also comments which do not belong to candidate of the fifth comment are removed except the fifth comment itself. Finally, formula (3) is used to calculate Ranking-distance score. Two comments which are the most similar to each other have more Ranking-distance score. In Figure 8, the most score belong to relation with the fourth comment. This feature is symmetric and the similarity among comments is only calculated one time. In other words, this feature needs *O*(*N* ^2^) time complexity for text similarity pairwise comparisons.

(3) $$\begin{array}{c} \text{Ranking-distance}\;\left(C1,C2\right)={\left(\left|C1\&C2\right|+\left|C2\&C1\right|\right)}^{-1}, \end{array}$$

where *C*1 and *C*2 are two comments and |*C*1&*C*2| is the text similarity distance between *C*1 and *C*2.

#### 4.2.6. Frequent Patterns of Authors Appearance

The idea is that the comments of two commenters who talk to each other, usually appear closely in chronological order. So, if their comments appear many times just after each other, this feature gives a high score to their comments. In order to implement this feature, we use the following formula:

(4) $$\begin{array}{c} \text{FPScore}\;\left(i,j\right)=f\left(A_{i},A_{j}\right)+f\left(A_{j},A_{i}\right), \end{array}$$

where *A*_*i*_ is the author of comment *i*, *f*(*a*, *b*) is the number of times of comments of author *a* that appear just before comments of author *b* (see Pseudocode 1).

**Figure 9:**
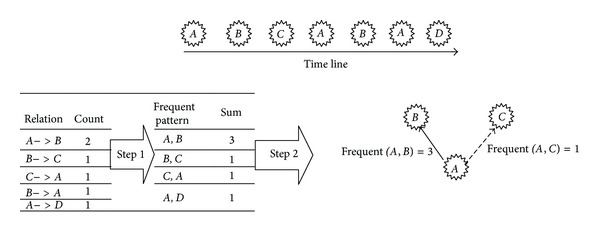
An example for authors appearance sequence.

Figure 9 shows a time line which includes 7 comments which have been written by 4 commenters. The feature score is calculated for the relation among comments. The score of relation between comments *A* and *B* is 3 which is more than *A* and *C*.

#### 4.2.7. Length Ratio

The length of the parent text is usually longer than its children. The length ratio score is calculated according to

(5) $$\begin{array}{c} \text{length}\;\text{ratio}\;\text{score}\;\left(C_{C},C_{P}\right)=\frac{\left|C_{P}\right|}{\left|C_{C}\right|}, \end{array}$$

where *C*_*C*_ is a comment looking for a parent and *C*_*P*_ is a candidate parent.

#### 4.2.8. Frequent Words in Parent-Child Comments

Sometimes a pair of words appears to be frequently one word in the parent and the next word in its children. For example in ENENews dataset, the most frequent pairs are (believe, people), (accident, fukushima), (new, discovered), (idea, report) and (people, public). We use pointwise mutual information (PMI) [38] to find the frequent pattern:

(6) $$\begin{array}{c} \text{Score}\;\left(W_{1},W_{2}\right)=\frac{\text{Count}\;\left(W_{1},W_{2}\right)}{\text{Count}\;\left(W_{1}\right)*\text{Count}\;\left(W_{2}\right)}, \end{array}$$

where *W*_1_ is a word in a comment whose parent we are looking for, *W*_2_ is a word in its candidate parent, and Count (*W*_1_, *W*_2_) is the number of time *W*_1_ has appeared in the parent and *W*_2_ has appeared in child. The numerator computes how often two words appear together and denominator computes how often one of the words appears. Finally, according to Pseudocode 2 the score of relation between two comments is calculated.

#### 4.2.9. Candidate Filtering 

In addition to the features, we use some heuristics to filter some inappropriate candidates.

1. Usually a commenter does not reply to the root in his/her second comment. So if a commenter has written more than one comment, the root is removed from the parent candidates of the second and next comment. This was shown to be a useful feature because the root is an important candidate, so if we could remove it correctly, the results are improved significantly.
2. A commenter does not reply to him/herself. So we simply remove all comments of a commenter from his/her comment's candidates [2].
3. Commenters who post only one comment on a thread are more likely to reply to the root post. So other candidates can be removed except the root.

## 5. Experiments

In this section, we provide details about the datasets used for evaluation, describe the evaluation metrics, and then present the results of the SLARTS method and compare them with the results of the baseline approaches.

### 5.1. Dataset

Unfortunately, there is no standard dataset for RTS task in declarative threads, while several datasets are available for interrogative threads [3, 4, 14]. Therefore, Thestandard (http://thestandard.org.nz/), Alef (http://www.alef.ir/), ENENews (http://enenews.com/), Russianblog (www.kavery.dreamwidth.org/), and Courantblogs (www.courantblogs.com/) websites were crawled to provide evaluation data. Thestandard in New Zealand and Alef in Iran are two online news agencies, established in August 2007 and 2006, respectively. They publish daily news and articles by journalists in different categories such as economy, environment, politics, and society. Thestandard is in English and Alef is in Persian. Thestandard has provided the tree-like reply structure of comments since 2010. ENENews is a news website covering the latest energy-related developments. It was established shortly after the Fukushima Daiichi disaster in March 2011. It has grown rapidly to serve approximately 2,000,000 page views per month. Russianblog and Courantblogs are two blogs that are in Russian and English, respectively. Russsianblog and Courantblogs are established since 2003 and 2012, respectively.

The reason for selecting the mentioned websites are (1) They support multilevel reply; (2) their users are active and articles usually get many comments; (3) author and time of comments are available; (4) they cover different contexts and languages (news agencies, cultural sites, and blogs in English, Russian, and Persian).

For each website, we crawled the webpages that were published until the end of 2012. We then parsed the pages and extracted the reply structure and used it for ground truth. We have removed the threads with less than 4 comments, because these kinds of threads do not give us much information and usually their comments reply to the root.  Table 1 summarizes the information about the prepared datasets. The datasets are available at http://ece.ut.ac.ir/nlp/resources.html.

In ENENews and Russianblog, users have to register in order to leave a comment. However, in other datasets, users can leave comments in a thread with different names or different users can use the same names.

Table 2 reports some statistics on the crawled websites. The length of comments' text in Russianblog is shorter than the other datasets which causes text similarity measures to perform poorly on it. In ENENews, the root usually includes a tweet, that is why the length of the root's text is shorter than the other datasets. All comments have author's name except for some comments in Alef. Therefore the numbers of commenters for Alef was calculated from the comments that have author's name. The average number of comments per article in Thestandard and ENENews are about 50 and 39, respectively, which are larger than the other datasets.

In order to gain some insights into the data, we show some charts extracted from the datasets.

**Figure 10:**
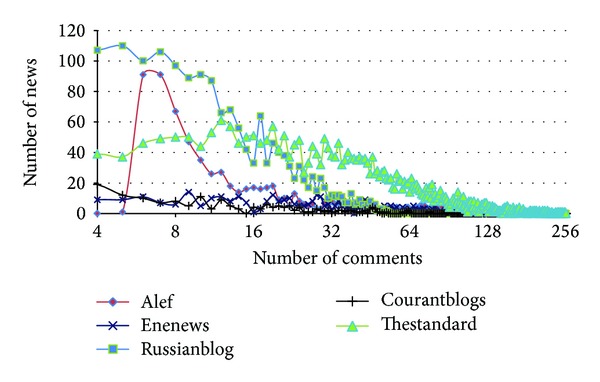
Distribution of the number of comments in news article.

Figure 10 shows the distribution of the number of comments in the articles. It is seen that most threads have between 5 and 15 comments in Russianblog and Alef. However, in Thestandard, length of threads is longer than the other datasets and most threads have between 12 and 24 comments.

**Figure 11:**
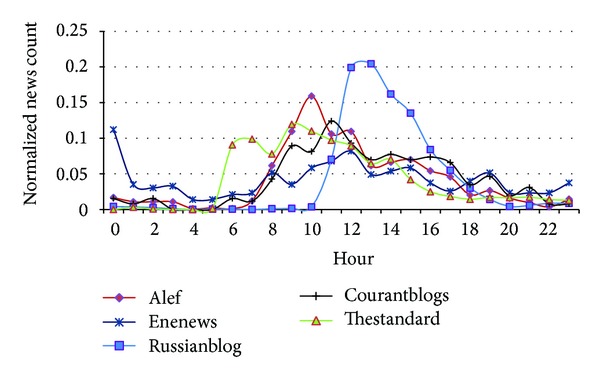
Publication rate of articles per hour.

Publication rate of articles is shown in Figure 11. The publication rate follows a bell-shape and articles are published between 7 am and 19 pm and the highest rate belongs to the 4-hour period between 9 am and 13 pm. Since there is only one author who publishes the articles in Russianblog, its chart has less variation and the root is usually published between 10 am and 18 pm.

**Figure 12:**
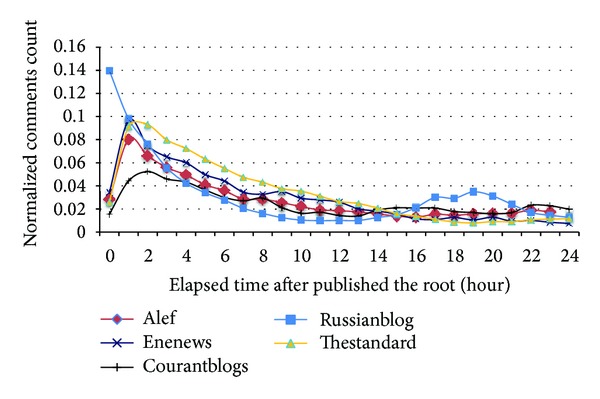
Average number of published comments per hour after the root.

Figure 12 shows the publication rate of comments after the root. It is seen that all datasets except Russianblog have similar behavior: about one hour after the article is published, it has received most of the comments. After that, the rate of comment reception decreases. Russianblog shows different behavior. It seems that a part of its visitors reply to its articles the next morning, 16–21 hours after the publication.

**Figure 13:**
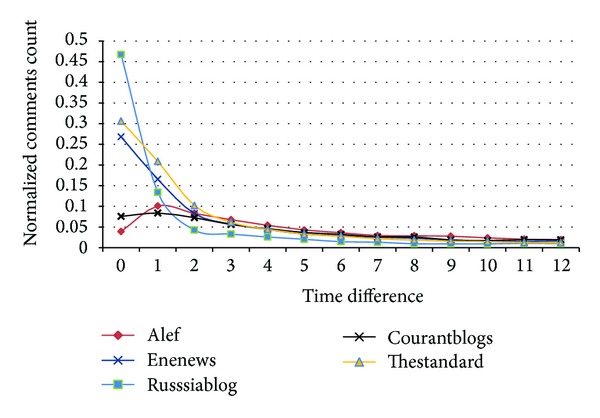
Time difference between a node and corresponding parent per hour.

Figure 13 shows the time difference between publication time of comments and their replies. It is seen that the maximum difference is usually less than one hour. ENENews and Russianblog do not moderate comments and Thestandard has very active moderators who immediately check and accept comments. However, in Courantblogs and Alef, where moderators are not always online, the time difference is between one and two hours.

**Figure 14:**
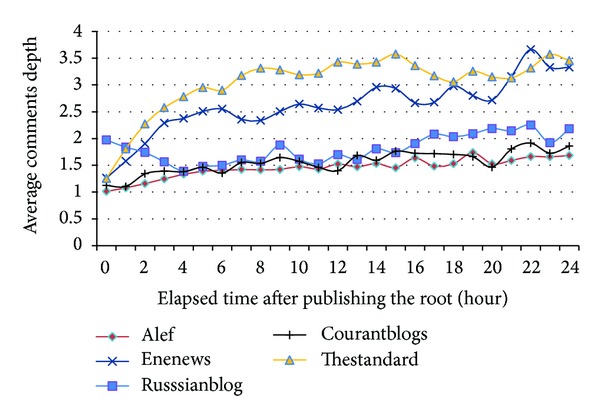
Depth of comments averaged on the elapsed time from publication of root.

Figure 14 shows how depth of comments increases when time passes after publication of the main article. Deeper comments show longer and more serious conversations. As shown in the figure, comments usually reply to the root in early hours. After a few hours, conversations are continued around the comments which causes the depth of thread to increase. Visitors of Alef and Courantblogs talk more about the root article. However, Thestandard and ENENews have longer and deeper discussions.

**Figure 15:**
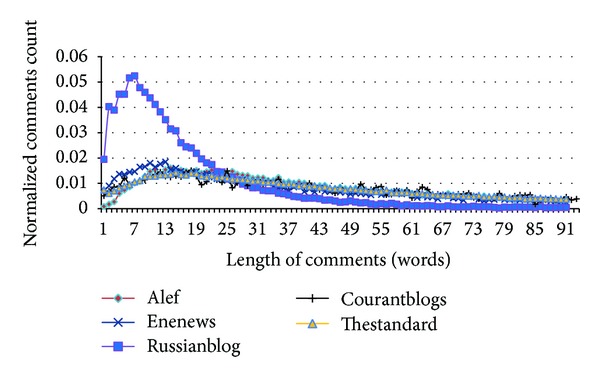
Histogram of length of comments in words.

Figure 15 shows length of comments in words. Most of comments include 10–20 words. Except for comments of Russianblog, the other datasets are similar. This tells that the similarity measure is not enough and we have to use nontextual features as well. Russianblog has comments that are shorter than the other datasets. This dataset is a personal blog and users usually write friendly comments.

**Figure 16:**
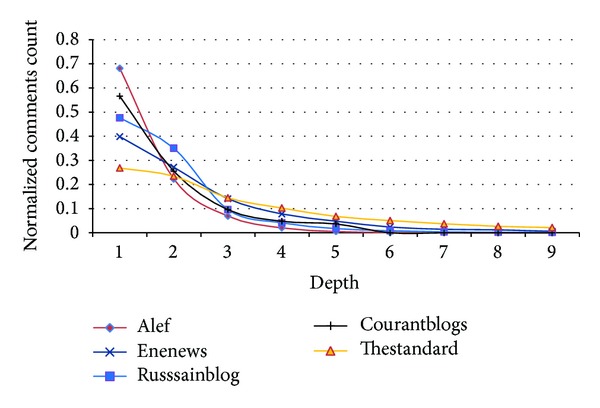
Histogram of the depth of comments.

Figure 16 shows, depth of comments. Depth is directly related to the conversations. It is seen that comments are usually found in the first depth (below the root). Russianblog, ENENews, and Thestandard have more comments in higher levels meaning conversations are longer.

### 5.2. Evaluation Metrics

To evaluate the experiment results, we use several metrics. For edge prediction we use precision, recall, and *F*-score measures [4, 13, 17, 18]. Output of RTS is a tree. This causes precision, recall, and *F*-score values to be equal [2], since FP (False Positive) and FN (False Negative) in precision and recall are always equal. Instead, we use the following edge accuracy measure:

(7) Accuracy (Acc)edge=|{reply  relations}∩{detected  relations}|N,

where the tree is comprised of *N* comments and one root.

The second metric is path accuracy which was introduced by Wang et al. [3]. This metric has a global view and considers paths from nodes to the root:

(8) $$\begin{array}{c} {\text{Accuracy}\;\left(\text{Acc}\right)}_{\text{path}}=\frac{\sum_{i=1}^{\left|C\right|}\left|{\text{path}}_{G}\left(i\right)={\text{path}}_{P}\left(i\right)\right|}{\left|C\right|}, \end{array}$$

where path_*G*_(*i*) is the ground-truth structure for *i*th comment and path_*P*_(*i*) is the predicted structure for it. |*C*| is the number of comments in a thread. If any irrelevant comment appears in the path, this metric considers it to be completely wrong. So, it is very strict. To relax this, Wang et al. introduced a metric that computes the overlap between the paths in ground truth and the predicted path:

(9) $$\begin{array}{c} {\text{Precision}\;(P)}_{R\text{-path}}=\frac{\sum_{i=1}^{\left|C\right|}\left(\left|{\text{path}}_{G}\left(i\right)\cap {\text{path}}_{P}\left(i\right)\right|/\left|{\text{path}}_{P}\left(i\right)\right|\right)}{\left|C\right|},\\ {\text{Recall}\;(R)}_{R\text{-path}}=\frac{\sum_{i=1}^{\left|C\right|}\left(\left|{\text{path}}_{G}\left(i\right)\cap {\text{path}}_{P}\left(i\right)\right|/\left|{\text{path}}_{G}\left(i\right)\right|\right)}{\left|C\right|}, \end{array}$$

where |path_*P*_(*i*)| is the number of comments in the prediction path of *i*th comment and |path_*G*_(*i*)| is the number of comments in the ground-truth path of *i*th comment. *F*1-score is the harmonic mean of precision and recall:

(10) $$\begin{array}{c} F1\text{-score}=2\times \frac{\text{Precision}\times \text{recall}}{\text{Precision}+\text{recall}}. \end{array}$$

**Figure 17:**
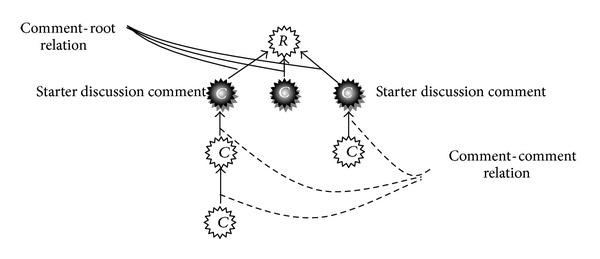
An illustration of a declarative thread.

The above mentioned metrics are appropriate in interrogative threads. As mentioned before, the root of declarative threads is news articles or main contents which are different from the root of interrogative threads. This causes the structure of threads and reply relations to be different. There are two types of reply relations in declarative threads: (1) comment-root, that is, the relation between comments and the root article and (2) comment-comment, that is, the relation between two comments, one parent, and one child. The comment-root relations show different views around the main article (root). The comment-comment relations show conversation among visitors which is a valuable source of information. In Figure 17, there are three comment-root and three comment-comment relations. When a user reads the root and comments, he/she can write his/her opinion or sentiment about the root by replying to root or participating in a discussion by replying to users' comments.

We believe that the evaluation metrics mentioned before are not enough to cover both types of reply relations due to differences between interrogative and declarative threads. An appropriate metric should be able to detect both types of relations. So, we have to modify the evaluation metrics or define new metrics.

We propose an evaluation metric, Accuracy (Acc)_CTD_, where CTD stands for Comments Type Detection. It is defined as the proportion of correctly detected comment types. The comment-root type includes comments which initiate a new view about the root (NP) and the comment-comment type includes comments which are part of a discussion (PD):

(11) $$\begin{array}{c} {\text{Accuracy}\;(\text{Acc})}_{\text{CTD}}=\frac{\text{TP}+\text{TN}}{\text{TP}+\text{TN}+\text{FP}+\text{FN}}, \end{array}$$

where TP is the number of correctly detected NP comments, TN is the number of correctly detected PD comments, FP is the number of incorrectly detected NP comments, and FN is the number of incorrectly detected PD comments.

To evaluate accuracy of comment-comment relations, the standard precision, recall, and *F*-score measures are used:

(12) $$\begin{array}{c} {\text{Precision}\;\left(P\right)}_{\text{edge}}=\frac{\text{TP}}{\text{TP}+\text{FP}\;},\\ {\text{Recall}\;\left(R\right)}_{\text{edge}}=\frac{\text{TP}}{\text{TP}+\text{FN}},\\ {F1\text{-score}\;\left(F\right)}_{\text{edge}}=2\times \frac{\text{Precision}\times \text{recall}}{\text{Precision}+\text{recall}}, \end{array}$$

where TP is the number of correctly detected comment-comment relations, FP is the number of incorrectly detected comment-comment relations, and FN is the number of comment-comment relations in the ground truth that were not predicted at all.

The path accuracy metric, mentioned earlier, is a modified version of *P*_path_ and *R*_path_ which is appropriate to declarative platform. This metric consider, discussion paths from each PD comment to the starter discussion comment but not to root:

(13) Precision (P)path={∑i=1|C|p|pathG(i)=pathP(i)||C|p,o.w.,1,if  (|C|p=0),Recall (R)path={∑i=1|C|R|pathG(i)=pathP(i)||C|R,o.w.,1,if  (|C|R=0),

where |*C*|_*R*_ is the number of PD comments in the ground-truth thread and |*C*|_*p*_ is the number of PD comments in the predicted thread. Path_*G*_(*i*) is the discussion path from *i*th node to the discussion starter comment in the ground truth. Path_*P*_(*i*) is discussion path from *i*th node to the discussion starter comment in the predicted thread.

Also, the relaxed Precision (*P*)_*R*-path_ and Recall (*R*)_*R*-path_ are modified to be suitable for declarative platform:

(14) Precision (P)R-path={∑i=1|C|p(|pathG(i)∩pathP(i)|/|pathP(i)|)|C|p,o.w.,1,if  (|C|p=0),Recall (R)R-path={∑i=1|C|R(|pathG  (i)∩pathP(i)|/|pathG(i)|)|C|R,o.w.,1,  if  (|C|R=0).

**Figure 18:**
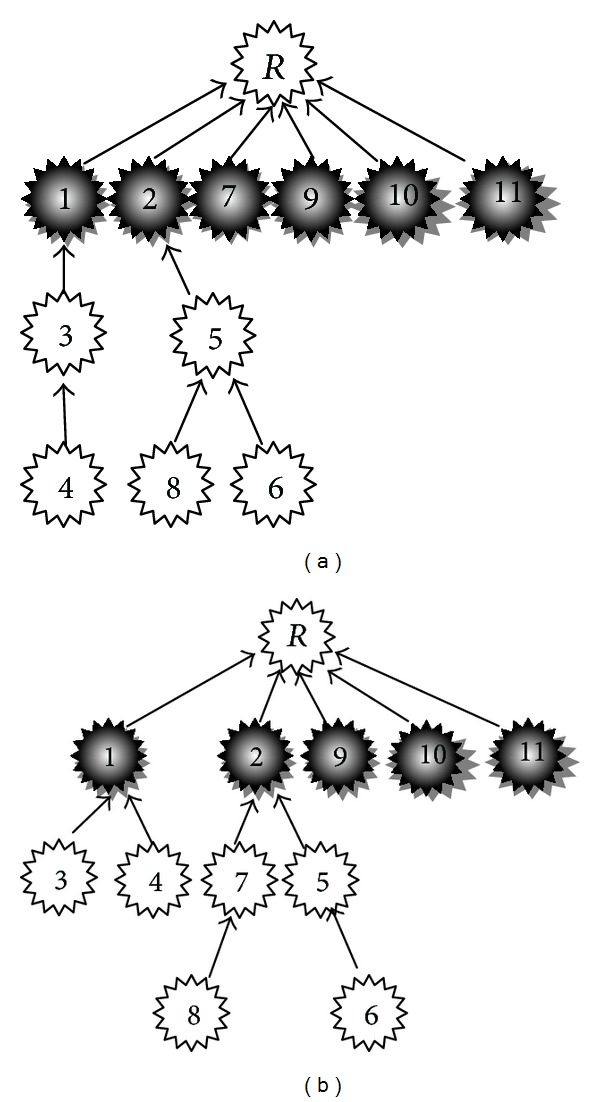
An example of two threads (a) ground-truth thread and (b) the prediction thread.

Figure 18 shows two threads of which one is the ground truth and the other one is the predicted thread. In order to better understand the evaluation metrics, we calculate them for this example.

In Table 3, the predicted thread in Figure 18 has been evaluated by metrics from interrogative threads. The results show high values.  Table 4 shows the results of evaluation by metrics from declarative threads. The results show that declarative metrics have more appropriate results.

There are two reasons which lead declarative metrics to better evaluate the predicted structure. (1) The root in declarative threads has many children. So, if a method connects all comments to the root, interrogative metrics show good results. (2) Interrogative metrics cannot properly evaluate comment-comment relations in declarative threads. However, the declarative metrics can evaluate both types of relations.

### 5.3. Experimental Results and Analysis

We use several baselines to compare the effectiveness of SLARTS. The first baseline is the performance when we simply link all comments to their previous comment (we name it Last baseline) [3, 4]. This method leads to good results for RTS task in interrogative threads. The second baseline is to link all comments to the root (we name it First baseline).

The only work that has been done on RTS has focused on the author's name in online news agencies [19]. Schuth et al.'s method used several features to find the commenter's name in online news articles. This method achieves a good precision but it has a low recall, because many comments do not refer to any author's name. So we selected Seo et al.'s method [2] which is a strong method in interrogative platforms.

Seo et al.'s method has focused on forums and emails and used the quoted text as one of their main features. This feature is not available in our datasets. Therefore, we use all their proposed features except the quoted text.

We have applied 5fold cross-validation to minimize bias with 95% as confidence interval.  Table 5 shows the results of our experiments based on interrogative evaluation metrics. According to Acc_edge_, SLARTS reveals higher accuracy except in Alef in which lots of replies are connected to the root.

According to *P*_*R*-path_ and *R*_*R*-path_, First and Last baselines have the best performance, respectively. First connects all comments to the root, this way irrelevant comments do not appear in the path from the comment to the root and also the root appears in all paths. Last connects all comments to their previous comments. This causes all comments to appear in the path.

According to Acc_path_, First has better performance in Thestandard, ENENews, and Alef, because more comments are linked directly to the root. Usually, SLARTS and Seo et al.'s methods cannot predict the full path in Thestandard and ENENews, because, according to Figure 15, paths are very long and complex in these datasets.

As we mentioned earlier, interrogative evaluation metrics are not appropriate for declarative threads, because based on these metrics, First shows high performance although this baseline does not detect any comment-comment relations.  Table 6 shows the results when we use declarative evaluation metrics proposed in Section 5.2.

According to Table 6, for *F*_edge_, SLARTS performs better than Seo et al.'s method. However, for *P*_edge_, Seo et al.'s method performs better but its *R*_edge_ is lower than SLARTS in all datasets.

It is important to say that, when the difference between *P*_edge_ and *R*_edge_ is high and *P*_edge_ is greater than *R*_edge_, the method most likely connects comments to the root and it does not appropriately detect comment-comment relations (like First baseline). On the other hand, when *R*_edge_ is greater than *P*_edge_, the method does not appropriately detect comment-root relations (like Last baseline).

So, Seo et al.'s method most likely connects comments to the root and this is not appropriately detecting comment-comment relations. On the other hands, *P*_edge_ and *R*_edge_ of SLARTS are close.

Several features in SLARTS such as Global similarity, frequent words, Frequent patterns, and authors' language focus on detection of comment-comment relations. This makes the results of SLARTS better than Seo et al.'s method in declarative evaluation metrics.

We already saw that the average length of threads in Thestandard and ENENews is longer than the other datasets (Table 2) and their paths are much longer and more complex than the other datasets (Figure 16). According to *F*_edge_, the accuracy of Thestandard and ENENews is less than other datasets. Note that *F*_edge_ is a strict metric in declarative threads.

SLARTS has better *F*_edge_ than Seo et al.'s method in all datasets. The maximum difference occurs in Thestandard and Russianblog. In these datasets many of the defined features have good performance. (Importance of features in ENENews and Russianblog datasets will be shown in Table 7 and we will explain more in the next section.)

As shown in Table 6, Russianblog has better results than the other datasets in all metrics. The main reason is that its comments are not confirmed by a moderator. This causes Acc_edge_ of Last baseline in Russianblog to be equal to 0.3632, that is, more than other datasets (Table 5). Acc_edge_ of Last baseline has inverse relationship with complexity of replies. Also, we showed in Figure 13 that the Russianblog has the lowest time difference between a comment and its parent. When time difference between a child and its parent decreases, detection of reply relation would be easier. In other words, as the parent appears to be closer to its child, some features such as frequent pattern and location Prior that are based on position of comments in chronological order work better.

The difference between *F*_*R*-path_  and *F*_path_ is about 20% in Thestandard and ENENews where threads are larger and paths have more complex structures.

The minimum difference between the results of SLARTS and Seo et al.'s methods appears in Alef datasets. In Alef many relations are comment root and many comments do not have author's name, which make the features perform poorly.

Since SLARTS method has features which specially focused on detecting comment-root relations (e.g., by adding candidate filtering), Acc_CTD_ of SLARTS is better than Seo et al. method in all datasets. The best result is 0.91 for Russianblog, and the worst result is 0.69 for ENENews. The root of ENENews dataset is usually a tweet. According to Table 2, it makes the average length of the root's text to be shorter than the other datasets and this makes the textual features perform poorly on detecting comment-root relations.

Confidence intervals of Alef and Courantblog datasets are higher than the other datasets, because many of their relations are comment root (Figure 16). This makes ranking SVM to be bias towards connecting comments to the root, especially when a thread includes very few comment-comment relations.

We compare *P*-value to specify the significance of differences between SLARTS and Seo et al.'s methods on declarative metrics. Since ranking SVM ranks candidates based on their score and selects the first candidate from the list, only the first candidate is important. So, p@1 is computed. The results indicate that all improvements are statistically significant (*P*-value < 0.005) in all datasets.

### 5.4. Evaluation of the Features

In this section, we evaluate the role of the introduced features. We use backward feature selection. That means to measure the importance of a feature we use all features except that feature, repeat the experiments in its absence, and compare the results to the case where all features are present. The difference between the values of metrics in presence of a feature and its absence is reported in Table 7.

It is seen that some features improve precision of the metrics, for example, location prior and candidate filtering rule 3, where they most likely tend to detect comment-root relations. Some features improve recall of the metrics such as authors' language, global similarity, candidate filtering rule 1, and frequent patterns. These features most likely tend to detect comment-comment relations. Some features affect both precision and recall, for example, authors' name.

As stated earlier, Russianblog has different behaviour in comparison with other datasets (Figures 12, 13, and 15). Also, ENENews has larger threads and more complex paths (Figure 16 and Table 2). So, we use these datasets to evaluate features in depth.

Similarity feature improves recall of evaluation metrics. Since Russian language is known as a morphologically rich language and the length of comments' text is very short (about average 18 words according to Table 2), in comparison with other datasets, improvement of textual features is low. To increase the performance of textual feature in Russian, we need a stemmer, a Wordnet, and a tokenizer. Similarity feature has rather a good impact on Acc_CTD_.

Authors' language feature improves recall of evaluation metrics. According to *R*_edge_, the improvements of this feature are 0.03 and 0.01 in ENENews and Russianblog, respectively.

Global similarity feature improves both precision and recall in Russianblog and recall in ENENews.

The frequent words feature has a small improvement in Russianblog like similarity feature. This feature improves recall of the evaluation metrics in ENEnews about 0.01.

Length ratio feature improves precision in both datasets. However, since longer comments in ENENews have many children, this feature is more prominent there.

Authors' name feature is useful in all evaluation metrics. The value of this feature in ENENews is more than Russianblog, because authors' name in Russianblog is different from other datasets; it is an email and no one would refer to it as a name.

The frequent patterns feature focuses on detecting comment-comment relations. This feature improves recall of evaluation metrics in both datasets.

The location prior feature improves precision in both datasets. This feature has a good improvement on Acc_CTD_. According to *P*_edge_, the best improvement is 0.16091 for Russianblog, since comments are not moderated.

The candidate filtering rule 1 improves recall of evaluation metrics in both datasets. This feature removes the root candidate accurately. This feature has a good improvement on Acc_CTD_. The maximum improvement for *R*_edge_ is 0.34 for Russianblog.

The candidate filtering rule 2 has small improvements on precision and recall of evaluation metrics in both datasets. Maximum improvement is gained with Russianblog.

Finally, The candidate filtering rule 3 improves precision of metrics in both datasets. This feature removes all candidates except the root. Thus, detection of comment-root relations is improved. Also, this feature improves Acc_CTD_.

## 6. Information Extraction by Visualizing Threads Structure

**Figure 19:**
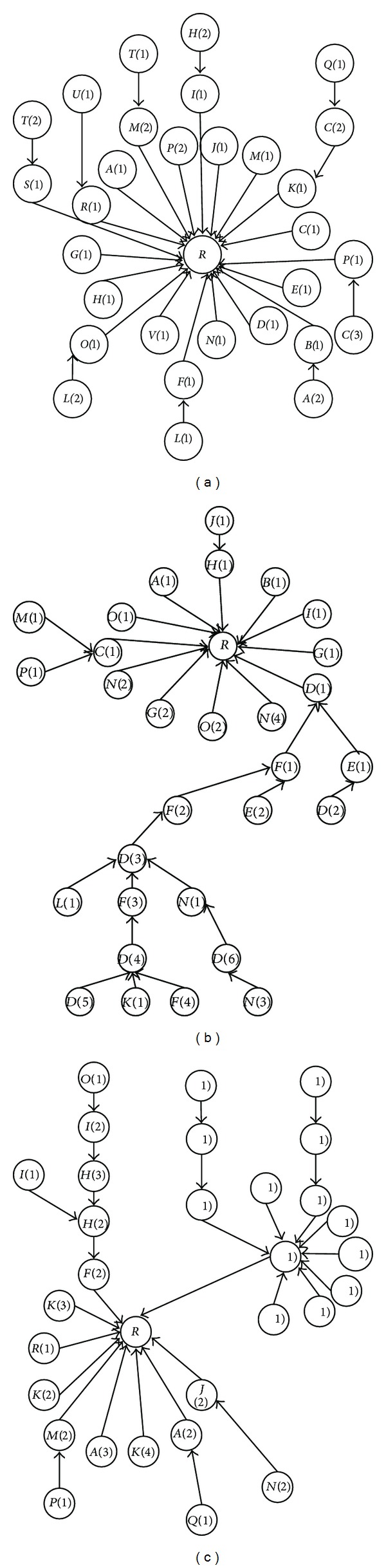
Three real threads in which nodes are labeled according to commenter's name and number of its comments.

In this section, we discuss the information which can be extracted from hierarchical structure of threads.  Figure 19 is the same as Figure 3 except that the commenters and their number of posted comments are shown as the nodes' label; for example, “*D*(3)” is the third comment sent by user “*D*”. Visualization of the structure reveals valuable information as following.

- *Is the root article controversial?* The threads “*B*” and “*C*” in Figure 19 are more controversial than “*A*”. Visitors have expressed different opinions and sentiments about the root in “*B*” and “*C*” leading to formation of longer conversations. The thread “*A*” shows a common thread which has short conversations about the root. The height and width of trees can help as a measure to recognize whether a root is controversial or not.
- *Which comments are starters [36]?* Starter comments have important role in the conversations because users read them and then read other comments to better understand the conversations.
- *Which comments have participated in this conversation?* Users can follow the conversations that seem interesting to them without reading any unrelated comment.
- *Who plays an important role in a discussion?* Some users play more important roles in conversations than other users. For example, users “*D*(1)” in thread “B” and “*A*(1)” in thread “*C*” have made a long conversation. These users are known as a hub or a starter in a thread and their degree of importance can be compared according to their indegree [39]. Many analyses can be performed on this structure which is known as popularity features [12].
- *How many conversations are about the root?* There are thirteen conversations which are replying directly to the root in thread “*C*,” for example, {F(2), H(2), H(3), I(2), O(1)} or {F(2), H(2), l(1)} show two conversations in thread “C”.

## 7. Conclusion and Future Work

In this paper, we proposed SLARTS, a method based on ranking SVM, which predicts the tree-like structure of declarative threads, for example, blogs and online news agencies. We emphasized on the differences between declarative and interrogative threads and showed that many of the previously proposed features perform poorly on declarative threads because of this. Instead, we defined a set of novel textual and nontextual features and used a ranking SVM algorithm to combine these features.

We detect two types of reply relations in declarative threads: comment-root relation and comment-comment relation. An appropriate method should be able to detect both types of reply relations and an appropriate metric should consider both types of reply relations. So, in order to have fair judge on the quality of the predicted structures, we modified the evaluation metrics accordingly. We also defined a novel metric that measures the accuracy of comment type detection.

The results of our experiments showed that, according to declarative evaluation metrics, our method shows higher accuracy in comparison with the baselines on five datasets. Also, we showed that all improvements in detecting comment-comment relations are statistically significant in all datasets.

We believe that the defined features are not limited to declarative threads. Some features such as author's language and frequent patterns extract relations among users can be useful in interrogative threads.

For future, we would like to use the proposed method on interrogative platforms such as forums. Also, we would like to analyze the tree-like reply structure deeply and we believe it can provide valuable information, for example, to help in understanding the role of users in discussions or to find important and controversial comments among a large volume of comments.

Since our goal is to provide a language-independent model to reconstruct the thread structure, we did not use text processing tools such as Wordnet and Named Entity Recognition (as they are not available in the same quality for all languages). We would like to focus on English and use these tools to find out whether they can improve the accuracy of RTS method or not.

## Figures and Tables

**Pseudocode 1:**
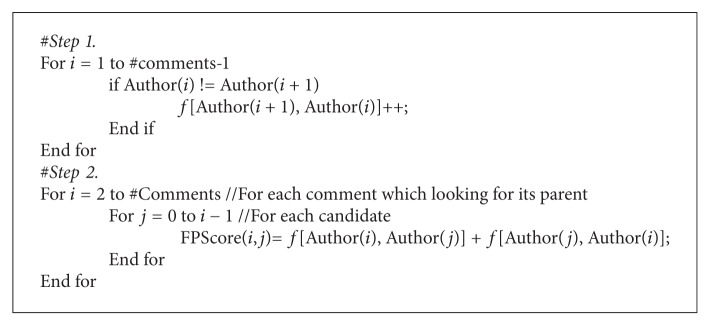
Pseudocode to calculate frequent patterns of authors appearance.

**Pseudocode 2:**
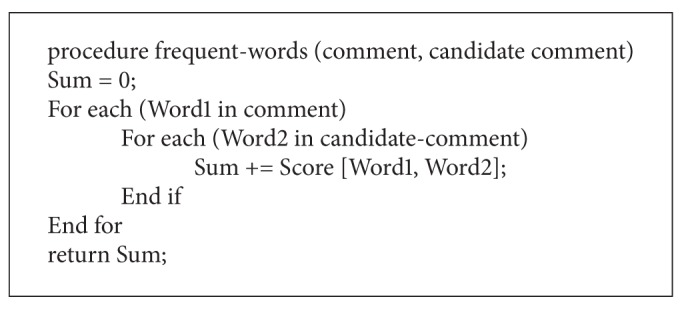
A pseudocode for calculating score of frequent words between two comments.

**Table 1:** The datasets specific properties.

Datasets	Language	Confirmation by moderator	Users register
Thestandard	English	*✓*	**×**
Alef	Persian	*✓*	**×**
ENENews	English	**×**	*✓*
Russianblog	Russian	**×**	*✓*
Courantblogs	English	*✓*	**×**

**Table 2:** Datasets' statistics split up per website.

Datasets	Avg. length of comments' text (in words)	Avg. length of the root's text (in words)	Number of comments	Avg. comments per news	Number of news	Number of commenter
Thestandard	72.98	357.68	149058	49.81	2992	3431
Alef	74.66	766.55	23210	27.96	830	4387
ENENews	68.19	162.64	16661	38.92	428	831
Courantblogs	79.24	398.17	4952	19.19	258	1716
Russianblog	18.56	237.16	30877	16.18	1908	1258

**Table 3:** Example of calculation of interrogative metrics for the example shown in Figure 18.

Evaluation metric	Calculation
Accuracy_edge_	Reply relations = {1 → R, 2 → R, 7 → R, 9 → R, 10 → R, 11 → R, 3 → 1, 4 → 3, 5 → 2, 6 → 5, 8 → 5} Detected relations = = {1 → R, 2 → R, 9 → R, 10 → R, 11 → R, 3 → 1, 4 → 1, 5 → 2, 6 → 5, 7 → 2, 8 → 7} $\frac{8}{11}\approx 0.73$

Acc_path_	$\frac{\left|\left(1\to R,2\to R,9\to R,10\to R,11\to R,3\to 1\to R,5\to 2\to R,6\to 5\to 2\to R\right)\right|}{\text{Number}\;\;\text{of}\;\;\text{comments}}=\frac{8}{11}\approx 0.73$

*P*_*R*-path_	$\frac{\left(1\to R,2\to R,9\to R,10\to R,11\to R,3\to 1\to R,5\to 2\to R,6\to 5\to 2\to R\right)+\left(4\to 1\to R,7\to 2\to R,8\to 7\to 2\to R\right)}{\text{Number}\;\;\text{of}\;\;\text{comments}}$ $=\frac{\left(1+1+1+1+1+1+1+1\right)+(1+1/2+2/3)}{11}=\frac{10.17}{11}\approx 0.92$

*R*_*R*-path_	$\frac{\left(1\to R,2\to R,9\to R,10\to R,11\to R,3\to 1\to R,5\to 2\to R,6\to 5\to 2\to R\right)+\left(4\to 3\to 1\to R,7\to R,8\to 5\to 2\to R\right)}{\text{Number}\;\;\text{of}\;\;\text{comments}}$ $=\frac{\left(1+1+1+1+1+1+1+1\right)+(2/3+1+2/3)}{11}=\frac{10.34}{11}\approx 0.94$

*F*1-score_*R*-path_	$\frac{2*0.92*0.94}{0.92+0.94}\approx 0.93$

**Table 4:** Example of calculation of declarative metrics for the example shown in Figure 18.

Evaluation metric	Calculation
Acc_CTD_	TP = {1, 2, 9, 10, 11} TN = {3, 4, 5, 6, 8} FN = {7} FP = { } $\frac{5+5}{5+5+1+0}\approx 0.91$

P_edge_ R_edge_ F1_edge_	TP = {3⟶1, 5⟶2, 6⟶5} $P_{\text{edge}}=\frac{3}{3+3}=0.5$ FN = {4⟶3, 8⟶5} $R_{\text{edge}}=\frac{3}{3+2}=0.6$ FP = {4⟶1, 7⟶2, 8⟶7} F1 − score_edge_ ≈ 0.55

*P*_path_ *R*_path_ *F*1_path_	$P_{\text{path}}=\;\frac{\left|\left\{3⟶1,5⟶2,6⟶5⟶2\right\}\right|}{\left|\left\{3⟶1,4⟶1,5⟶2,6⟶5⟶2,7⟶2,8⟶7⟶2\right\}\right|}=\frac{3}{6}=0.5$ $R_{\text{path}}=\;\frac{\left|\left\{3⟶1,5⟶2,6⟶5⟶2\right\}\right|}{\left|\left\{3⟶1,4⟶3⟶1,5⟶2,6⟶5⟶2,8⟶5⟶2\right\}\right|}=\frac{3}{5}=0.6$ *F*1-score_*R*-path_ ≈ 0.55

*P*_*R*-path_ *R*_*R*-path_ F1_R-path_	$P_{R\text{-path}}=\frac{\left\{3⟶1,4⟶1,5⟶2,6⟶5⟶2,7⟶2,8⟶7⟶2\right\}}{\left|\left\{3,4,5,6,7,8\right\}\right|}=\frac{1+1+1+1+0+1/2}{6}=\frac{4.5}{6}=0.75$ $R_{R\text{-path}}=\frac{\left\{3⟶1,4⟶3⟶1,5⟶2,6⟶5⟶2,8⟶5⟶2\right\}}{\left|\left\{3,4,5,6,8\right\}\right|}=\frac{1+1/2+1+1+1/2}{5}=\frac{4}{5}=0.8$ *F*1-${\text{score}}_{R\text{-path}}=\frac{2*0.75*0.8}{0.75+0.8}\approx 0.77$

**Table 5:** Experimental results with interrogative evaluation metrics.

Datasets	Methods	Acc_edge_	Acc_path_	*P*_*R*-path_	*R*_*R*-path_	F1_*R*-path_
Thestandard	SLARTS	**0.4669** **±0.009**	0.3059 **±**0.013	0.6927 **±**0.018	0.7450 ±0.010	0.7179 ±0.013
	Seo et al.'s [2]	0.3941 **±**0.005	0.3413 ±0.006	0.8303 ±0.009	0.6563 **±**0.005	0.7331 ±0.003
	First	0.3630 **±**0.010	**0.3630** **±0.010**	**1.0000** **±0.000**	0.5846 **±**0.009	**0.7379** **±0.007**
	Last	0.1824 **±**0.006	0.0495 **±**0.004	0.2214 **±**0.008	**1.0000** **±0.000**	0.3625 **±**0.010

ENENews	SLARTS	**0.4837** **±0.018**	0.3695 **±**0.027	0.7625 ±0.026	0.7770 ±0.013	0.7697 **±**0.017
	Seo et al.'s [2]	0.4661 **±**0.017	0.4134 ±0.021	0.8720 ±0.011	0.7086 ±0.013	0.7818 ±0.011
	First	0.4524 **±**0.018	**0.4524** **±0.018**	**1.0000** **±0.000**	0.6634 ±0.016	**0.7976** **±0.011**
	Last	0.2090 **±**0.016	0.0596 **±**0.003	0.2319 ±0.011	**1.0000** **±0.000**	0.3764 **±**0.015

Courantblogs	SLARTS	**0.6843** **±0.028**	0.6453 **±**0.033	0.9127 ±0.013	0.8619 ±0.014	0.8865 **±**0.013
	Seo et al.'s [2]	0.6700 **±**0.040	**0.6628** **±0.043**	0.9667 ±0.014	0.8250 ±0.018	**0.8902** **±0.015**
	First	0.6490 **±**0.036	0.6490 **±**0.036	**1.0000** **±0.000**	0.8001 ±0.019	0.8889 **±**0.012
	Last	0.2037 **±**0.017	0.1082 **±**0.008	0.3183 ±0.011	**1.0000** **±0.000**	0.4829 **±**0.012

Russianblog	SLARTS	**0.7463** **±0.017**	**0.6904** **±0.017**	0.8741 ±0.008	0.8787 ±0.006	**0.8764** **±0.007**
	Seo et al.'s [2]	0.5446 **±**0.010	0.5174 **±**0.011	0.9293 ±0.003	0.7821 ±0.004	0.8494 **±**0.003
	First	0.4968 **±**0.006	0.4968 **±**0.006	**1.0000** **±0.000**	0.7134 ±0.004	0.8327 **±**0.003
	Last	0.3632 **±**0.018	0.1549 **±**0.012	0.3695 ±0.015	**1.0000** **±0.000**	0.5395 **±**0.016

Alef	SLARTS	0.7753 **±**0.017	0.7641 **±**0.018	0.9579 ±0.006	0.9033 ±0.008	0.9298 **±**0.007
	Seo et al.'s [2]	0.7802 ±0.019	0.7800 ±0.019	0.9950 ±0.003	0.8828 ±0.010	0.9355 ±0.006
	First	**0.7853** **±0.018**	**0.7853** **±0.018**	**1.0000** **±0.000**	0.8826 ±0.010	**0.9376** **±0.006**
	Last	0.0993 **±**0.005	0.0822 **±**0.005	0.2526 ±0.012	**1.0000** **±0.000**	0.4032 **±**0.015

The bold style shows the best result in each metric.

**Table 6:** Experimental results with declarative evaluation metrics.

Datasets	Method	*P*_edge_	*R*_edge_	F_edge_	*P*_path_	*R*_path_	*F*_path_	*P*_*R*-path_	*R*_*R*-path_	F_R-path_	Acc_CTD_
Thestandard	SLARTS	**0.3782** **±0.008**	**0.4155** **±0.011**	**0.3960** **±0.006**	**0.1565** **±0.007**	**0.1713** **±0.008**	**0.1636** **±0.007**	**0.3628** **±0.012**	**0.4166** **±0.016**	**0.3876** **±0.005**	**0.7525** **±0.005**
	Seo et al.'s [2]	0.3014 ±0.031	0.1414 ±0.015	0.1921 ±0.016	0.1544 ±0.025	0.0576 ±0.011	0.0835 ±0.013	0.3013 ±0.038	0.1319 ±0.018	0.1831 ±0.021	0.5831 ±0.007
	Difference	0.0778	0.2741	0.2039	0.0021	0.1137	0.0801	0.0615	0.2847	0.2045	0.1694

ENENews	SLARTS	0.3797 ±0.038	**0.3891** **±0.019**	**0.3839** **±0.024**	0.1873 ±0.040	**0.1855** **±0.026**	**0.1857** **±0.030**	0.3768 ±0.048	**0.3768** **±0.017**	**0.3759** **±0.025**	**0.6872** **±0.014**
	Seo et al.'s [2]	**0.4060** **±0.030**	0.1713 ±0.016	0.2407 ±0.018	**0.2378** **±0.034**	0.0837 ±0.016	0.1235 ±0.021	**0.3947** **±0.035**	0.1523 ±0.015	0.2196 ±0.019	0.5954 ±0.009
	Difference	−0.0263	0.2178	0.1432	−0.0505	0.1018	0.0622	−0.0179	0.2245	0.1563	0.0918

Courantblogs	SLARTS	0.5236 ±0.030	**0.3963** **±0.073**	**0.4495** **±0.052**	0.4220 ±0.057	**0.3110** **±0.058**	**0.3566** **±0.049**	0.5325 ±0.049	**0.3853** **±0.075**	**0.4452** **±0.057**	**0.7625** **±0.033**
	Seo et al.'s [2]	**0.7320** **±0.090**	0.2049 ±0.062	0.3189 ±0.082	**0.6815** **±0.105**	0.1896 ±0.070	0.2951 ±0.093	**0.7309** **±0.094**	0.2010 ±0.065	0.3140 ±0.086	0.7001 ±0.031
	Difference	−0.2084	0.1914	0.1306	−0.2595	0.1214	0.0615	−0.1984	0.1843	0.1312	0.0624

Russianblog	SLARTS	**0.5940** **±0.028**	**0.5674** **±0.022**	**0.5803** **±0.023**	**0.4907** **±0.031**	**0.4664** **±0.020**	**0.4782** **±0.024**	**0.5878** **±0.028**	**0.5823** **±0.021**	**0.5849** **±0.020**	**0.9116** **±0.011**
	Seo et al.'s [2]	0.4705 ±0.020	0.3030 ±0.015	0.3685 ±0.016	0.3823 ±0.023	0.2534 ±0.013	0.3046 ±0.015	0.4682 ±0.020	0.2822 ±0.014	0.3521 ±0.015	0.5862 ±0.013
	Difference	0.1235	0.2644	0.2118	0.1084	0.213	0.1736	0.1196	0.3001	0.2328	0.3254

Alef	SLARTS	0.6189 ±0.028	**0.4131** **±0.040**	**0.4952** **±0.037**	0.5639 ±0.027	**0.3819** **±0.037**	**0.4551** **±0.034**	0.6277 ±0.027	**0.4069** **±0.043**	**0.4934** **±0.039**	**0.8044** **±0.013**
	Seo et al.'s [2]	**0.8827** **±0.052**	0.2637 ±0.051	0.4045 ±0.060	**0.8797** **±0.053**	0.2631 ±0.051	0.4034 ±0.060	**0.8840** **±0.052**	0.2635 ±0.051	0.4043 ±0.060	0.7846 ±0.018
	Difference	−0.2638	0.1494	0.0907	−0.3158	0.1188	0.0517	−0.2563	0.1434	0.0891	0.0198

The bold style shows the best result in each metric.

**Table 7:** The difference between the evaluation metrics in presence and absence of features.

Feature	Dataset	Evaluation metric						
		*P*_edge_	*R*_edge_	*P*_Path_	*R*_Path_	*P*_*R*-path_	*R*_*R*-path_	Acc_CTD_
Similarity	ENENews	−0.00233	0.07415	−0.00538	0.03289	−0.01095	0.08996	0.04684
	Russianblog	0.00874	0.00012	0.00963	0.00347	0.00695	−0.01079	0.00355

Authors' language	ENENews	−0.01354	0.03246	−0.01785	0.00693	−0.02757	0.04052	0.00458
	Russianblog	−0.00229	0.01052	−0.00179	0.00750	−0.00590	0.00807	−0.00182

Global similarity	ENENews	−0.00402	0.05604	−0.01338	0.01581	−0.01129	0.07086	0.02742
	Russianblog	0.01090	0.01443	0.00582	0.00897	0.00368	−0.00317	−0.00087

Frequent words	ENENews	0.00252	0.00931	0.00170	0.00424	−0.00132	0.00973	0.00292
	Russianblog	0.00008	0.00023	0.00016	0.00029	0.00034	0.00047	−0.00011

Length ratio	ENENews	0.04206	−0.02984	0.04450	0.01700	0.06421	−0.05336	−0.00850
	Russianblog	0.00651	−0.00355	0.00920	0.00080	0.00784	−0.00592	0.00235

Authors' name	ENENews	0.03753	0.06392	0.01920	0.03285	0.02341	0.05998	0.01788
	Russianblog	0.00000	0.00000	0.00000	0.00000	0.00000	0.00000	0.00000

Frequent pattern	ENENews	−0.02028	0.02930	−0.02357	0.00300	−0.03793	0.03410	0.00526
	Russianblog	0.00458	0.06100	0.00830	0.05206	−0.00545	0.06150	0.00404

Location prior	ENENews	0.08778	−0.03690	0.08556	0.03280	0.11792	−0.07852	0.02925
	Russianblog	0.16091	0.09671	0.17766	0.13515	0.16699	0.08793	0.02414

Candidate filtering rule 1	ENENews	−0.05431	0.06138	−0.04102	0.01296	−0.06458	0.07545	0.06949
	Russianblog	−0.12506	0.34370	−0.13916	0.27445	−0.12683	0.37003	0.32546

Candidate filtering rule 2	ENENews	0.00014	−0.00004	0.00048	0.00041	−0.00038	−0.00035	−0.00009
	Russianblog	0.02575	0.02475	0.02462	0.02341	0.01746	0.00802	0.00000

Candidate filtering rule 3	ENENews	0.00357	−0.00121	0.00452	0.00254	0.00551	−0.00161	0.00161
	Russianblog	0.08521	−0.01978	0.09636	0.01702	0.09380	−0.03825	0.07371
